# A deep learning reconstruction framework for X-ray computed tomography with incomplete data

**DOI:** 10.1371/journal.pone.0224426

**Published:** 2019-11-01

**Authors:** Jianbing Dong, Jian Fu, Zhao He

**Affiliations:** 1 Research Center of Digital Radiation Imaging and Biomedical imaging, Beijing University of Aeronautics and Astronautics, Beijing, China; 2 School of Mechanical Engineering and Automation, Beijing University of Aeronautics and Astronautics, Beijing, China; 3 Jiangxi Research Institute, Beijing University of Aeronautics and Astronautics, Nanchang, China; Zhejiang University, CHINA

## Abstract

As a powerful imaging tool, X-ray computed tomography (CT) allows us to investigate the inner structures of specimens in a quantitative and nondestructive way. Limited by the implementation conditions, CT with incomplete projections happens quite often. Conventional reconstruction algorithms are not easy to deal with incomplete data. They are usually involved with complicated parameter selection operations, also sensitive to noise and time-consuming. In this paper, we reported a deep learning reconstruction framework for incomplete data CT. It is the tight coupling of the deep learning U-net and CT reconstruction algorithm in the domain of the projection sinograms. The U-net estimated results are not the artifacts caused by the incomplete data, but the complete projection sinograms. After training, this framework is determined and can reconstruct the final high quality CT image from a given incomplete projection sinogram. Taking the sparse-view and limited-angle CT as examples, this framework has been validated and demonstrated with synthetic and experimental data sets. Embedded with CT reconstruction, this framework naturally encapsulates the physical imaging model of CT systems and is easy to be extended to deal with other challenges. This work is helpful to push the application of the state-of-the-art deep learning techniques in the field of CT.

## Introduction

The invention of X-ray computed tomography (CT) has led to a revolution in many fields such as medical imaging, nondestructive testing and materials science. It can overcome the limit from radiographic imaging, namely that three-dimensional objects are projected on a two-dimensional plane and the depth information becomes invisible. Employing a set of two-dimensional projections to reconstruct the three-dimensional objects, CT allows us to investigate the inner structures in a quantitative and nondestructive way.

Image reconstruction plays an important role during the development of CT and many reconstruction algorithms have been proposed over the last decades. Filtered back projection (FBP) is generally preferred since it keeps a good balance between reconstruction speed and image quality when applied to complete data. However, limited by implementation conditions, CT with incomplete projections happens quite frequently. For example, the projections in the well-known sparse-view and limited-angle CT are incomplete [1–5]. The corresponding FBP reconstruction will have quite visible artifacts and noise.

Reconstruction with incomplete projections has attracted more and more interests. Using the Kaczmarz method in numerical linear algebra, Gordon et al gave out an algebraic reconstruction technique (ART) [6] for the direct CT reconstruction from a few projections. Good results could be obtained by updating each image pixel with the deviation between the measured projections and the simulated forward projections. However, it works in a ray by ray mode and is hard to suppress noise. Anderson et al proposed simultaneous ART (SART) [7] in which the updating is executed after the deviations of all rays are calculated out. The reconstructed image becomes smooth and the stripe-shaped artifacts are better suppressed. The maximum likelihood expectation maximization (MLEM) [8] algorithm proposed by Shepp and Vardi was also applied to image reconstruction. Involved with the statistical properties of X-ray photons, it is effective to reduce the imaging dose. Another popular algorithm is penalized likelihood reconstruction [9]. With prior knowledge, it performs well to improve the low dose resolution. Based on the compressed sensing theory, Sidky et al developed total variation (TV) techniques [10] to deal with incomplete projections. Wang et al introduced a new TV minimization method for limited-angle CT reconstruction [1]. Their method needs less time to obtain better results than other TV methods. Hu et al presented an improved statistical iterative algorithm for sparse-view and limited-angle CT [2]. Using penalized weighted least-squares criteria for TV minimization and including a feature refinement step to recover features lost in the TV minimization, their results show significant improvement over other iterative algorithms in terms of the preservation of structural details and the suppression of undesired patchy artifacts. Luo et al proposed an image reconstruction method based on TV and wavelet tight frame for limited-angle CT problem [11]. They used SART and TV to get the initial result. Then a hard thresholding method was utilized to cut the smaller wavelet coefficients. Lastly, the Lagrange multiplier method was used to update the dual variable. It further improves the quality of reconstructed images. These reconstruction techniques could be better than FBP, but they still have some limits such as expensive time consumption for the successive iterative steps and the complicated parameter selection.

A more recent trend is the application of deep learning (DL). It has led to a series of breakthroughs for image classification [12, 13] and segmentation [14] and also demonstrated impressive results on signal denoising [15] and artifacts reduction [16, 17]. DL naturally takes into account low, middle and high level features [18] and the “levels” of features can be enriched by increasing the number of stacked layers (depth). Driven by the significance of depth, an notorious obstacle, vanishing/exploding gradients [19, 20], has arisen. It hampers the network to converge. This problem has been addressed by normalized initialization [21–23] and intermediate normalization layers [24]. However, with the network’s depth increasing, accuracy gets saturated and then degrades rapidly as reported in [25, 26]. He et al has introduced a deep residual learning theory [27] to address this problem, with which it would be easier to push the residual to be zero than to fit an identity mapping by a stack of nonlinear layers.

DL has recently been applied to X-ray CT reconstruction. Cierniak combined a back-projection operation with a hopfield neural network to reconstruct CT image from projections [28]. It fixed parameters in the back-projection and used neural network to recover information that was blurred during the back-projection. Würfl et al directly mapped FBP onto a deep neural network architecture and demonstrated that image reconstruction can be expressed in terms of neural networks [29]. Based on a persistent homology analysis, Han et al developed a deep learning residual architecture [30] for sparse-view CT reconstruction. The input of this architecture is the initial corrupted CT image from FBP or other algorithm. It firstly estimates topologically simpler streaking artifacts from the input image and then subtracts the estimated result from the input image to get artifact-free image. Obviously this method is independent on X-CT reconstruction and works in an indirect mode. Using multi-scale wavelet, they extended their work to limited angle CT reconstruction [31]. Jin et al also proposed a deep convolutional neural network [32] for inverse problem in imaging. It is similarly independent on X-CT reconstruction, but the estimated results are the final CT images not the artifacts. With dilated convolutions, Pelt et al introduced an architecture [33] to capture features at different image scales and densely connect all feature maps with each other. Their method is also independent on CT reconstruction, but has the ability to achieve accurate results with fewer parameters. It reduced the risk of overfitting the training data. Yang et al presented a deep convolutional neural network (CNN) method that increases the acquired X-ray tomographic signal during the low-dose fast acquisition by improving the quality of recorded projections. Short-exposure-time projections enhanced with CNNs show signal-to-noise ratios similar to long-exposure-time projections. However, it could not suppress the artifacts caused by sparse-view or limited-angle scanning. The reported results have demonstrated that these approaches are much faster than the conventional algorithms and also easier to be implemented.

In this paper, we report a new deep learning reconstruction framework for X-CT with incomplete projections. Different from above mentioned techniques, it is the tight coupling of the deep learning U-net [14] and FBP algorithm in the domain of the projection sinograms. The estimated result are not the CT image or the artifacts, but the complete projection sinograms. Embedded with CT reconstruction, it naturally encapsulates the physical imaging model of CT systems and is easy to be extended to deal with other challenges such as beam-hardening, scattering and noise. When training, this framework firstly obtains the forward projections from the initial reconstruction by applying FBP to the original incomplete projection sinograms. Taking the complete projection sinograms as a target, they are then input into the U-net to obtain the net parameters by doing deep learning. After training, this framework is determined and can obtain the final CT image from a given incomplete projection sinogram. Taking the sparse-view and limited-angle CT as examples, this framework has been validated by using synthetic data sets and experimental data sets. This work is helpful to push the application of deep learning in the field of CT.

## Methods

### Proposed framework

**Fig 1:**
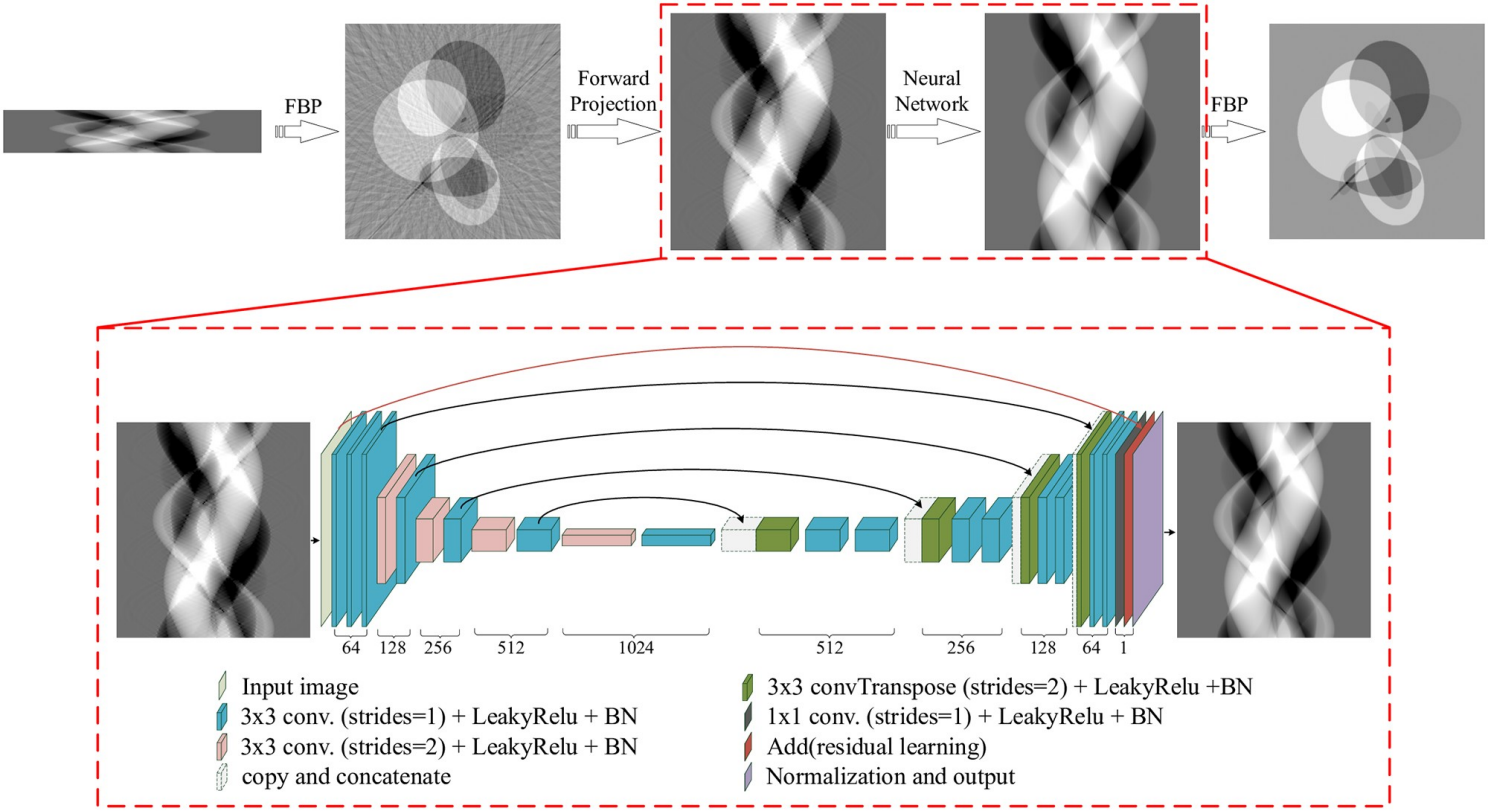
The architecture of the proposed deep learning reconstruction framework for X-CT with incomplete projections. For each three-dimensional block, the height and width represent the feature map’s size and the depth corresponds to the number of channels in this feature map.

Fig 1 shows the proposed deep learning reconstruction framework for X-CT with incomplete projections. It is based on FBP and the deep learning U-net and called DLFBP. This framework consists of four parts. Initial FBP reconstruction of the original incomplete sinogram is the first part. The second part is the forward projection operator which is applied to the initial reconstructed image to obtain corrupted sinogram. The convolutional neural network U-net is the third part and used to execute deep learning. The final FBP reconstruction with the complete sinogram from the third part is the last part of the framework. Obviously, the third part is the iconic one and works in the domain of sinograms.

Eq (1) is the well-known two-dimensional equi-spaced fan beam FBP algorithm which is adopted to reconstruct the CT image in this article. It also has many extension versions for different CT scanning configurations. In this equation, *β*(*x*, *y*) represents the CT image in Cartesian coordinate system, *U* the geometrical weight factor, *P*(*w*, *ϕ*) the projection sinogram, *h* the inverse Fourier transform of the ramp filter, *w* the index of the detector channels and *ϕ* the CT rotation angle. The symbol “*” means convolution.

$$\begin{array}{c} \beta (x,y)=\frac{1}{2}\int_{0}^{2\pi }U\times P(w,\phi )*h(w)d\phi \end{array}$$ (1)

Eq (2) is the forward projection operator. In this equation, *P*(*w*, *ϕ*) represents the forward projection recorded by the *w*th detector channel at the rotation angle *ϕ*, *β*(*x*, *y*) the initial reconstructed CT image and *l* the forward projection path.

$$\begin{array}{c} P(w,\phi )=\int_{-\infty }^{+\infty }\beta (x,y)dl \end{array}$$ (2)

Eqs (3) and (4) formulate the U-net. In these two equations, *f*() represents the extractor to recognize and characterize the context from input *X* in the encoding way, Λ[] the nonlinear mapping function, *F*{} the constructor to obtain part of output $\hat{Y}$, ***W*** and *bias* the parameters trained and determined in the neural network. One can refer to [14] for more details.

$$\begin{array}{c} \hat{Y}=F\{\Lambda [f(X)]\}+X \end{array}$$ (3)

$$\begin{array}{c} f(X)=W^{T}\cdot X+bias \end{array}$$ (4)

The parameter ***W*** is a vector and trained with the following steps. Firstly, it is initialized with a Gaussian distribution with standard deviation $\sqrt{\frac{2}{n_{in}+n_{out}}}$ in which *n*_*in*_ and *n*_*out*_ indicate the number of input and output units in each layer. Then, for each pair of training data which consists of original input *X* and its corresponding ground truth *Y*, the mean square error *E* between $\hat{Y}$ and *Y* is calculated out with Eq (5), where *m* represents the number of samples in one batch used for training. Finally *E* is used to update ***W*** via backward propagation expressed in Eq (6). *t* represents the number of the iteration training, *j* the parameter index in the vector ***W*** and *η* the learning rate. When the learning rate *η* is big, the parameters in the network are updated a lot. It is helpful to reach convergence fast. However, learning oscillation may happen if it is too big. In implementation, gradually reducing the learning rate is a good idea.

$$\begin{array}{c} E=\frac{1}{2m}{\left(Y-\hat{Y}\right)}^{2} \end{array}$$ (5)

$$\begin{array}{c} w_{j}^{t+1}=w_{j}^{t}-\eta \cdot \frac{\partial E}{\partial w_{j}^{t}} \end{array}$$ (6)

The parameter *bias* is also trained and determined by using the above method. When the mean square error *E* reaches the convergence condition, the training stops and a determined network is available for work.

### Convolutional filter for down-sampling

**Fig 2:**
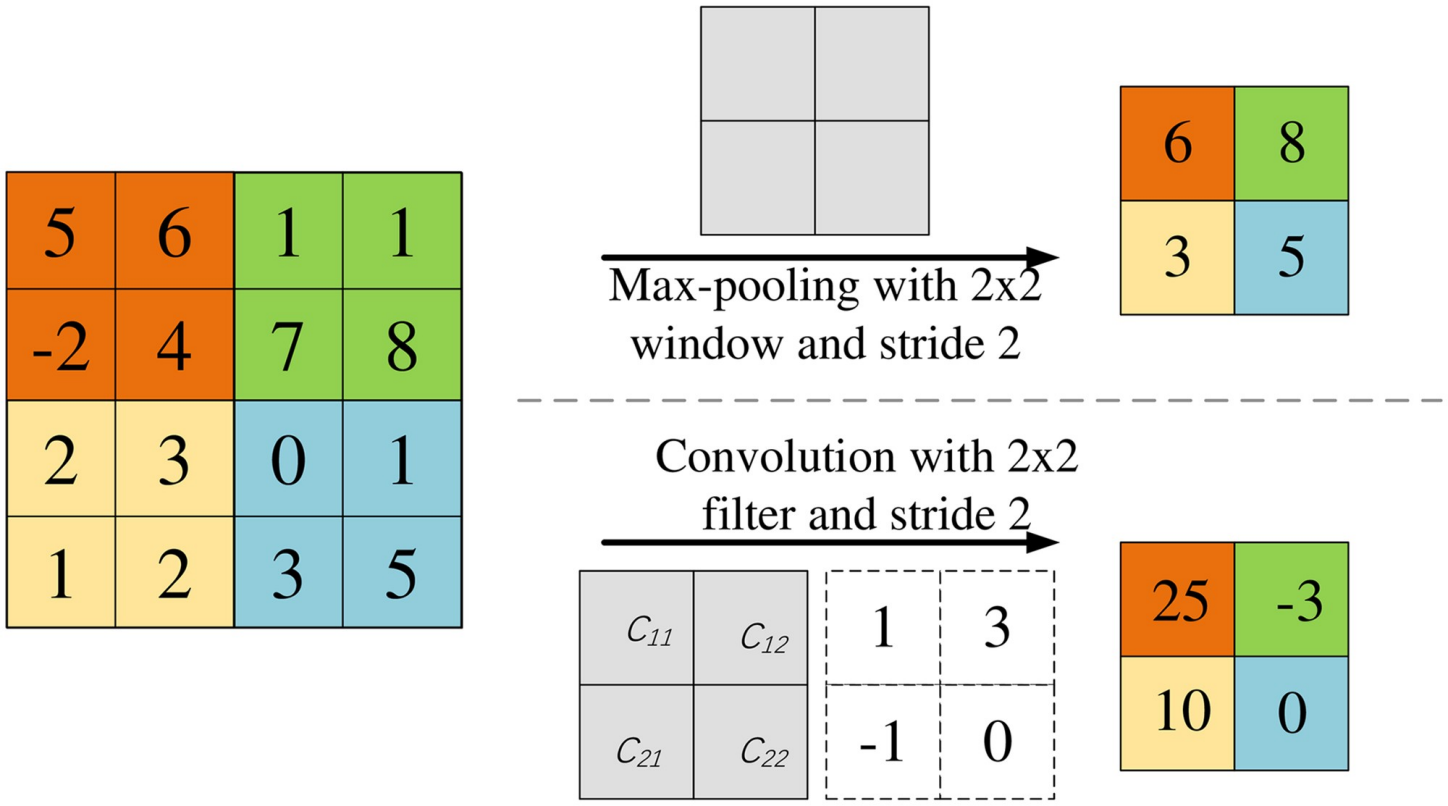
The working principles of the max-pooling operator and the convolutional filter.

Down-sampling is one of the fundamental operations within U-net and implemented by the so-called max-pooling operator. It is mainly used to reduce the dimensions of the feature maps and increase the size of the receptive field. The pooling size should keep balance between these two factors. The determination relies on experience and the size usually set to be 2. The stride is correspondingly set to be 2 to obtain the same dimension reduction. As an example, the max-pooling with a 2 × 2 window and a stride 2 is depicted in Fig 2. At each window, max-pooling extracts the maximum value and removes others. It runs fast, but some details may be lost in this processing. We adopt a convolution filter with a 2 × 2 window and a stride 2, shown in Fig 2, to replace the max-pooling. All values in the window will make their contributions to the down-sampling according to the four window coefficients *C*_11_, *C*_12_, *C*_21_ and *C*_22_. This new operation will improve the down-sampling accuracy since these four window coefficients are updated iteratively during the training.

### Normalization

This framework involves complicated calculations such as image reconstruction, forward projection and convolution. They may lead to unignored computation errors and degrade the training efficiency and accuracy. So we apply normalization operation to input *X*, output $\hat{Y}$ and ground truth *Y* to avoid this problem. This normalization operation is expressed in Eq (7) in which *I*_*n*_ represents the normalized image, *I* the raw image, *mean*() an operator to obtain mean value and *std*() an operator to obtain standard deviation value.

$$\begin{array}{c} I_{n}=\frac{I-mean(I)}{std(I)} \end{array}$$ (7)

### Running modes

This framework has two running modes. One is training mode and the other is working mode. Training mode has following steps: i) A set of incomplete sinograms is firstly matched with the corresponding complete sinograms into many pairs of training data. Each pair consists of an incomplete sinogram and a complete sinogram. ii) These data is input into the framework depicted in Fig 1 one pair by one pair and the network parameters such as ***W***, *bias*, *C*_11_, *C*_12_, *C*_21_ and *C*_22_ are updated iteratively. iii)When all pairs are used once, an outer learning iteration is completed. iv) Repeat steps ii) and iii) until the learning converges.

The procedure for working mode is much simpler. When an incomplete sinogram is fed into the framework determined by the training mode, the output of the framework will be a high quality CT image.

### Layer parameters

For the following experiments, the learning framework in Fig 1 has totally 27 lays. Table 1 lists the corresponding parameters of these layers. In this table, layer indexes start with the input layer indicated by the light green block in Fig 1.

**Table 1:** The values of the parameters in the deep learning layers.

Layers	Input Size	Output Size	Stride Size	Kernel Size
1: Input	720 × 731 × 1	720 × 731 × 1	—	—
2: Conv	720 × 731 × 1	720 × 731 × 64	1 × 1	3 × 3
3: Conv	720 × 731 × 64	720 × 731 × 64	1 × 1	3 × 3
4: Conv	720 × 731 × 64	720 × 731 × 64	1 × 1	3 × 3
5: Conv	720 × 731 × 64	360 × 366 × 128	2 × 2	2 × 2
6: Conv	360 × 366 × 128	360 × 366 × 128	1 × 1	3 × 3
7: Conv	360 × 366 × 128	180 × 183 × 256	2 × 2	2 × 2
8: Conv	180 × 183 × 256	180 × 183 × 256	1 × 1	3 × 3
9: Conv	180 × 183 × 256	90 × 92 × 512	2 × 2	2 × 2
10: Conv	90 × 92 × 512	90 × 92 × 512	1 × 1	3 × 3
11: Conv	90 × 92 × 512	45 × 46 × 1024	2 × 2	2 × 2
12: Conv	45 × 46 × 1024	45 × 46 × 1024	1 × 1	3 × 3
13: ConvTranspose	45 × 46 × 1024	90 × 92 × 512	2 × 2	3 × 3
14: Conv	90 × 92 × (512 + 512)	90 × 92 × 512	1 × 1	3 × 3
15: Conv	90 × 92 × 512	90 × 92 × 512	1 × 1	3 × 3
16: ConvTranspose	90 × 92 × 512	180 × 183 × 256	2 × 2	3 × 3
17: Conv	180 × 183 × (256 + 256)	180 × 183 × 256	1 × 1	3 × 3
18: Conv	180 × 183 × 256	180 × 183 × 256	1 × 1	3 × 3
19: ConvTranspose	180 × 183 × 256	360 × 366 × 128	2 × 2	3 × 3
20: Conv	360 × 366 × (128 + 128)	360 × 366 × 128	1 × 1	3 × 3
21: Conv	360 × 366 × 128	360 × 366 × 128	1 × 1	3 × 3
22: ConvTranspose	360 × 366 × 128	720 × 731 × 64	2 × 2	3 × 3
23: Conv	720 × 731 × (64 + 64)	720 × 731 × 64	1 × 1	3 × 3
24: Conv	720 × 731 × 64	720 × 731 × 64	1 × 1	3 × 3
25: Conv	720 × 731 × 64	720 × 731 × 1	1 × 1	3 × 3
26: Add	720 × 731 × (1 + 1)	720 × 731 × 1	—	—
27: Norm	720 × 731 × 1	720 × 731 × 1	—	—

## Experiments

Sparse-view CT and limited-angle CT are two typical cases with incomplete data. Taking them as examples, this section validates the proposed reconstruction framework. Two experiments were executed. The first one is based on synthetic data set. Some of the synthetic data was used to train the framework and others to test. The second is based on real data set. Some of the real data was used to train the framework and others to test.

### Data preparation

For synthetic data sets, 300 phantoms are used to obtain the fan beam sinograms with different sampling factors. Each phantom consists of tens of ellipses with random attenuation coefficients, sizes, and locations. In sparse-view CT experiments, the sampling factors are set to be 1, 8 and 12. They correspond to 720, 90 and 60 views respectively. The size of each phantom is 512 × 512 pixels. Fan beam sinograms are generated by using the embedded MATLAB function *fanbeam*(). The width of all the sinograms are 731 pixels. The sinogram with sampling factor 1 has a size 720 × 731 pixels and is treated as a complete one. Other sinograms are incomplete. Sinograms of 200 phantoms are used to train the framework and those from another 100 phantoms are used to test the framework.

Within the framework, for each incomplete sinogram, the initial FBP reconstruction is firstly executed with Eq (1) to obtain the initial CT image. Then the forward projection operator in Eq (2) is applied to the initial CT image to generate the corresponding corrupted sinogram with a size 720 pixels × 731 pixels. Next the iterative deep learning runs to update the network parameters by making comparison between the corrupted sinogram and the complete sinogram.

For experimental data sets, 300 female and male head slice images are randomly chosen from Visible Human Project CT Datasets. 200 images are used to train the network and the left 100 images are used to test the network. All the operations and procedure are the same as the ones for synthetic data sets.

The experimental data sets are available from https://www.nlm.nih.gov/research/visible/visible_human.html. A license agreement for this work was approved by the National Library of Medicine, National Institute of Health, USA.

### Implementation

This framework is implemented with Python 3.5.2 and Tensorflow 1.8. It runs in a workstation Advantech AIMB-785 with a CPU i7 6700 and a Graphics Processing Unit (GPU) nVidia GTX 1080Ti 11 GBytes.

Nadam optimizer [34] is used for the back propagation of gradients and updating learning parameters. Batch normalization (BN) [24] is adopted to accelerate the training. Due to the limit of GPU memory, the batch size set to be 1. It makes the training process unstable. In order to avoid the fluctuation of gradients that may affect the learning effect, a small learning rate is preferred to control the update of parameters. The initial learning rate is set to be 1 × 10^−4^ and gradually reduced with the exponential decay method expressed by Eq (8) from https://tensorflow.google.cn/versions/r1.10/api_docs/python/tf/train/exponential_decay. The decay rate is 10% and the decay steps 20. Int() represents the operation to obtain the integer division.

$$\begin{array}{c} decayed\;learning\;rate=learning\;rate\times decay\;rate^{int(global\;step/decay\;steps)} \end{array}$$ (8)

The training time for synthetic and experimental data sets is about 9 hours for 50 outer iterations. When testing, it takes 1*s* to obtain the final CT image.

### Image evaluation

Peak signal-to-noise ratio (PSNR) is a term for the ratio between the maximum possible power of a signal and the power of corrupting noise that affects the fidelity of its representation. Expressed in Eqs (9) and (10), it is commonly used to measure the quality of reconstruction of lossy compression codecs. In this article it is adopted to evaluate the quality of the sinograms and CT images. Here, *MAX*() is an operator to obtain the maximum value, *m* and *n* the height and width of the image, *G* the ground truth image and *K* the image to be evaluated. The bigger PSNR, the better the image quality.

The averaged PSNR (aPSNR) value is also used to evaluate the performance of the proposed framework. It is the mean value of a group of PSNR values and calculated by Eq (11) in which *num* is the number of PSNR values.

$$\begin{array}{c} PSNR=20\cdot \log_{10}\left(\frac{MAX(G)}{\sqrt{MSE}}\right) \end{array}$$ (9)

$$\begin{array}{c} MSE=\frac{1}{mn}\sum_{i=0}^{m-1}\sum_{j=0}^{n-1}{\left\|G(i,j)-K(i,j)\right\|}^{2} \end{array}$$ (10)

$$\begin{array}{c} aPSNR=\frac{1}{num}\sum_{i=1}^{num}PSNR_{i} \end{array}$$ (11)

### Comparison with methods based on CT images

As mentioned in the Introduction section, most of the existing X-CT image deep learning processing techniques are independent on CT reconstruction algorithms. The input is the corrupted CT image, and the output is the corrected CT image or artifact. In contrast, the proposed method is the combination of CT reconstruction algorithms and deep learning techniques, and works in the domain of the projection sinograms. The estimated results are the complete sinograms. It has the potential to provide much better image quality.

A comparison between the proposed method and a typical deep convolution neural network(DCNN) [32] was conducted to demonstrate its performance. During the implementation of DCNN, all the procedures and the parameters which are set manually and not determined by the training, kept the same as those published in the reference [32].

## Results

**Fig 3:**
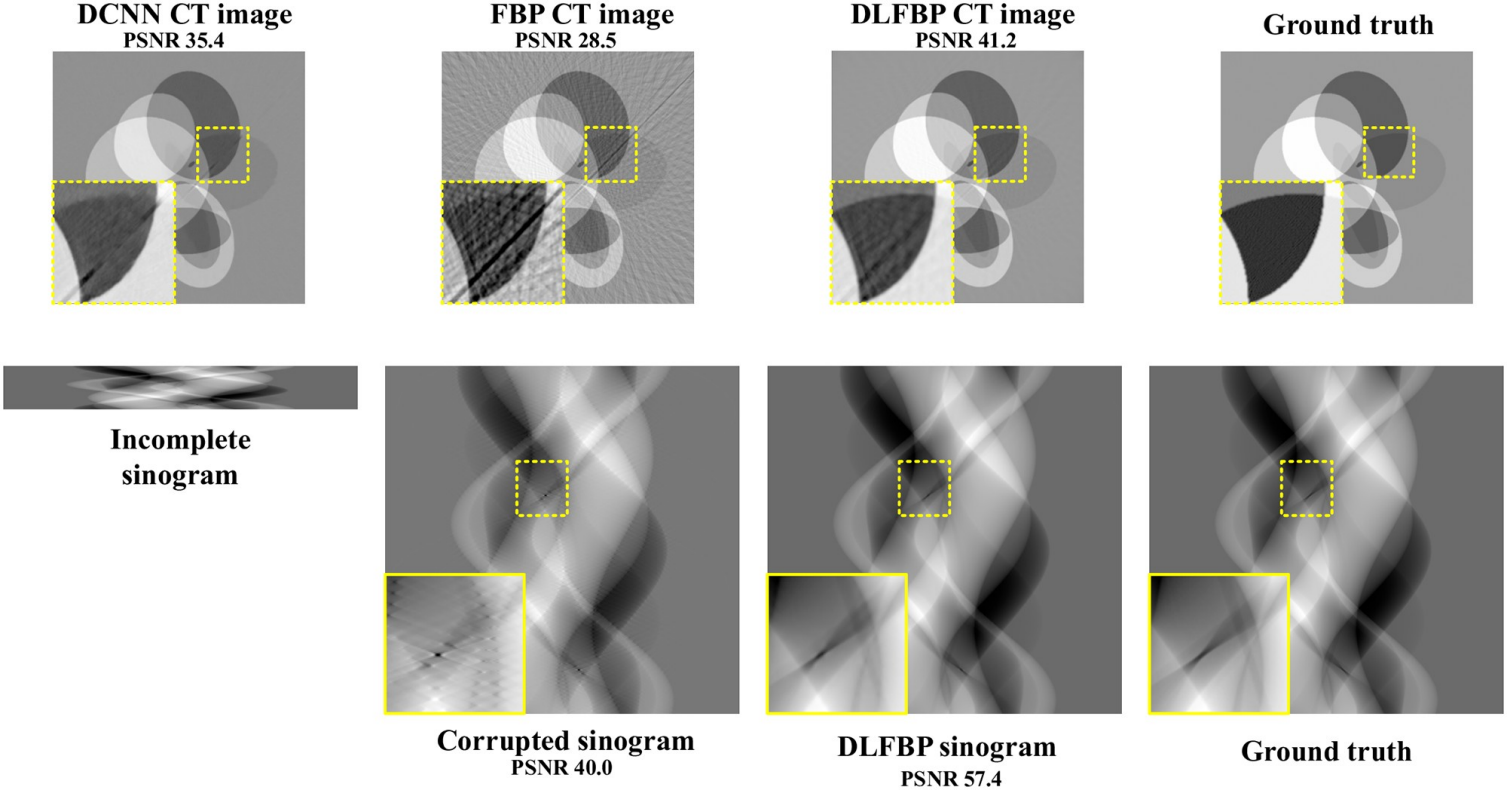
The sparse-view results of one synthetic phantom with an incomplete sinogram with 90 views by using DCNN [32], FBP and DLFBP. The incomplete singoram has a size of 90 × 731 pixels. The corrupted sinograms generated by the forward projection have a size of 720 × 731 pixels. All CT images have a size of 512 × 512 pixels after cutting off the surrounding blank region. Some regions of these images, indicated by the yellow box, are enlarged for better visualization.

**Fig 4:**
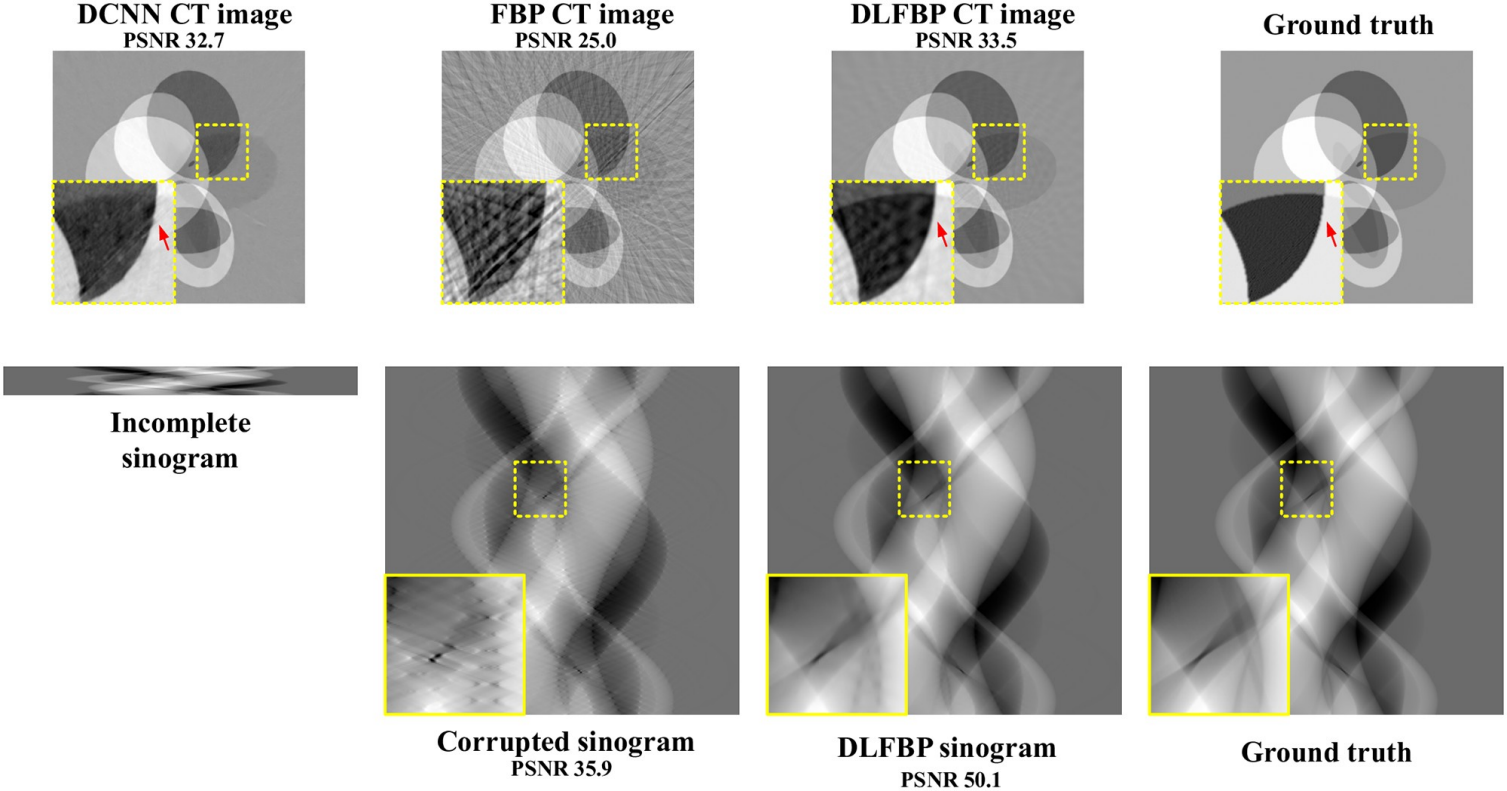
The sparse-view results of one synthetic phantom with an incomplete sinogram with 60 views by using DCNN [32], FBP and DLFBP. The incomplete singoram has a size of 60 × 731 pixels. The corrupted sinograms generated by the forward projection have a size of 720 × 731 pixels. All CT images have a size of 512 × 512 pixels after cutting off the surrounding blank region. Some regions of these images, indicated by the yellow box, are enlarged for better visualization.

**Fig 5:**
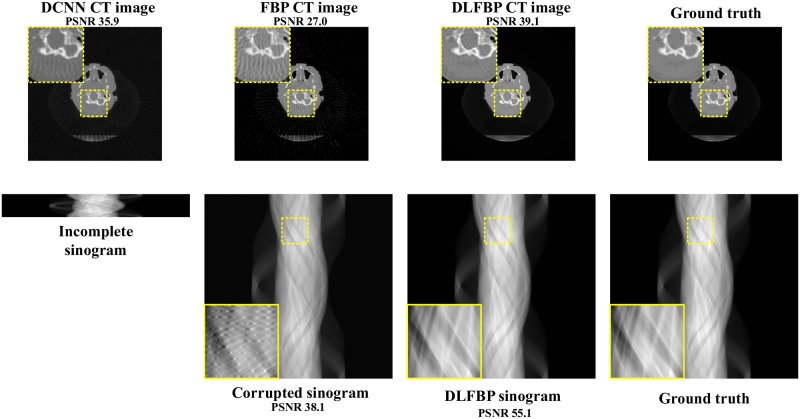
The sparse-view results of one head slice with an incomplete sinogram with 90 views by using DCNN [32], FBP and DLFBP. The incomplete singoram has a size of 90 × 731 pixels. The corrupted sinograms generated by the forward projection have a size of 720 × 731 pixels. All CT images have a size of 512 × 512 pixels after cutting off the surrounding blank region. Some regions of these images, indicated by the yellow box, are enlarged for better visualization.

**Fig 6:**
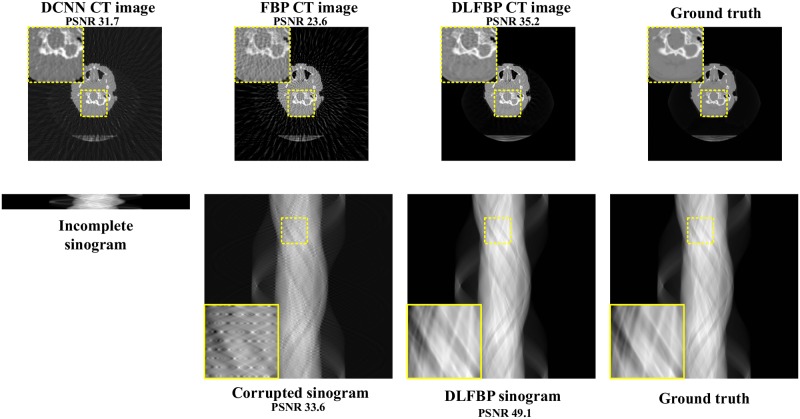
The sparse-view results of one head slice with an incomplete sinogram with 60 views by using DCNN [32], FBP and DLFBP. The incomplete singoram has a size of 60 × 731 pixels. The corrupted sinograms generated by the forward projection have a size of 720 × 731 pixels. All CT images have a size of 512 × 512 pixels after cutting off the surrounding blank region. Some regions of these images, indicated by the yellow box, are enlarged for better visualization.

Figs 3 and 4 present the sparse-view results of one of the 100 synthetic phantoms for testing. Figs 5 and 6 present the sparse-view results of one of the 100 head slices for testing. They correspond to the cases with sampling factors of 8 and 12, respectively. Their incomplete singorams have sizes of 90 × 731 pixels and 60 × 731 pixels, respectively. All the corrupted sinograms generated by the forward projection have a size of 720 × 731 pixels. All the reconstructed CT images have a size of 512 × 512 pixels after cutting off the surrounding blank region. In order to show the most obvious difference among each group of images, some regions of interest (ROIs) in these images, indicated by the yellow boxes, are enlarged for better visualization. In these four figures, it is noticeable that, after training, the artifacts caused by the sparse sampling are suppressed drastically both in the sinograms and CT images. The PSNR values also significantly increase. They demonstrate the validity of the proposed framework.

**Fig 7:**
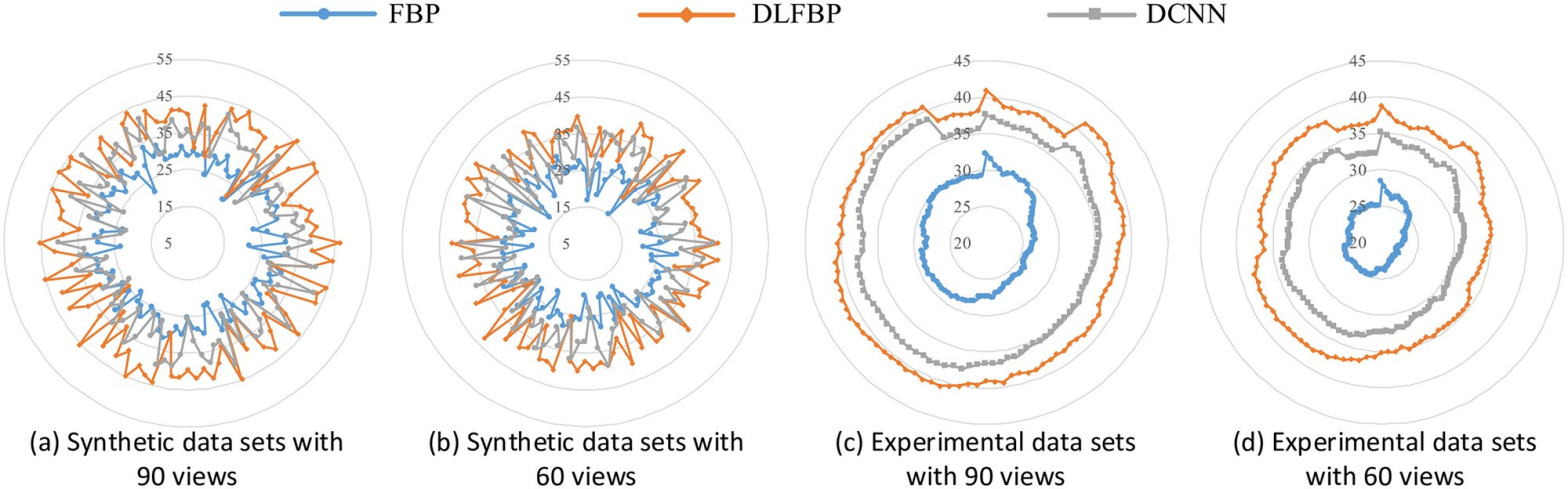
The PSNR values of the sparse-view CT images. The blue points with circle masks present the PSNR values of the CT images reconstructed by FBP. The orange points with rhombus masks present the PSNR values of the results by DLFBP. The gray points with square masks present the PSNR values of the results by DCNN [32].

Fig 7 shows the radar maps of the PSNR values of all the 100 synthetic and experimental images for sparse-view CT testing. Table 2 lists the corresponding aPSNR values. They also confirm the validity of the proposed framework.

**Table 2:** aPSNR values of sparse-view results.

Cases		Corrupted Sinogram	DLFBP Sinogram	FBP CT Image	DCNN CT Image	DLFBP CT Image
Synthetic aPSNR(dB)	90 views	38.17	53.29	28.42	33.28	39.01
	60 views	33.99	46.47	25.25	30.81	35.52
Experimental aPSNR(dB)	90 views	38.25	53.15	28.22	36.75	40.22
	60 views	33.49	49.30	24.43	32.68	37.21

It should be pointed that although DCNN significantly reduces the sparse sampling artifacts, some of them remain in the images. Moreover, the bigger the sampling factor is, the more blurry the edges of the object are. These may be caused by the fact that its learning mechanism is based on CT images. Once an imprecise value is estimated by the DCNN, it will directly distort the object in the final CT image. In contrast, DLFBP is based on sinograms. An estimation error in sinograms will be compensated by the following CT reconstruction since the final CT is a weighted sum of the values in sinograms. As such, DLFBP is tolerant to learning bias and provides better image quality.

**Fig 8:**
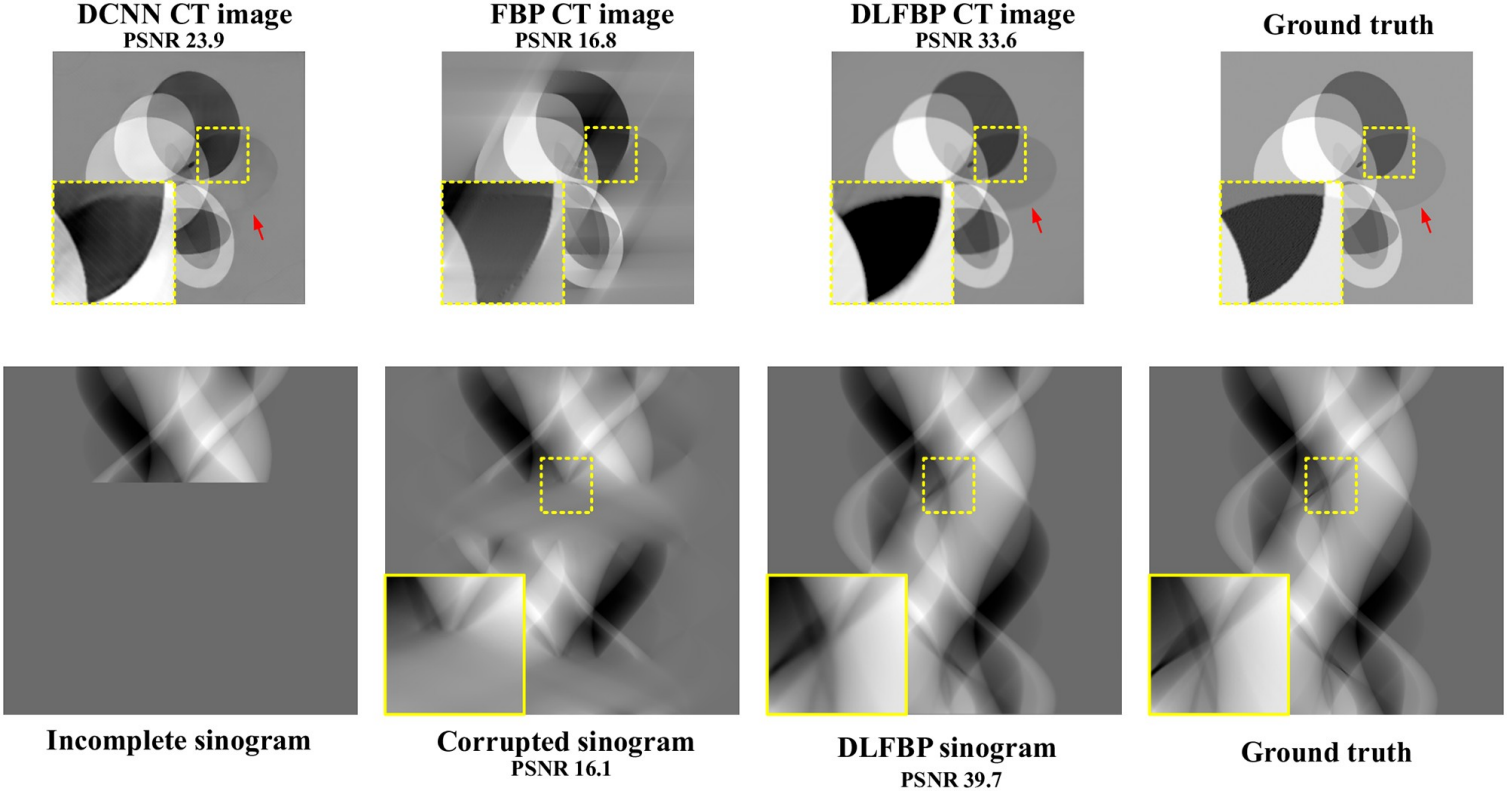
The limited-angle results of one synthetic phantom with the projections within the angular range [0 120°] by using DCNN [32], FBP and DLFBP. The incomplete singoram has a size of 240 × 731 pixels. The corrupted sinograms generated by the forward projection have a size of 720 × 731 pixels. All CT images have a size of 512 × 512 pixels after cutting off the surrounding blank region. Some regions of these images, indicated by the yellow box, are enlarged for better visualization.

**Fig 9:**
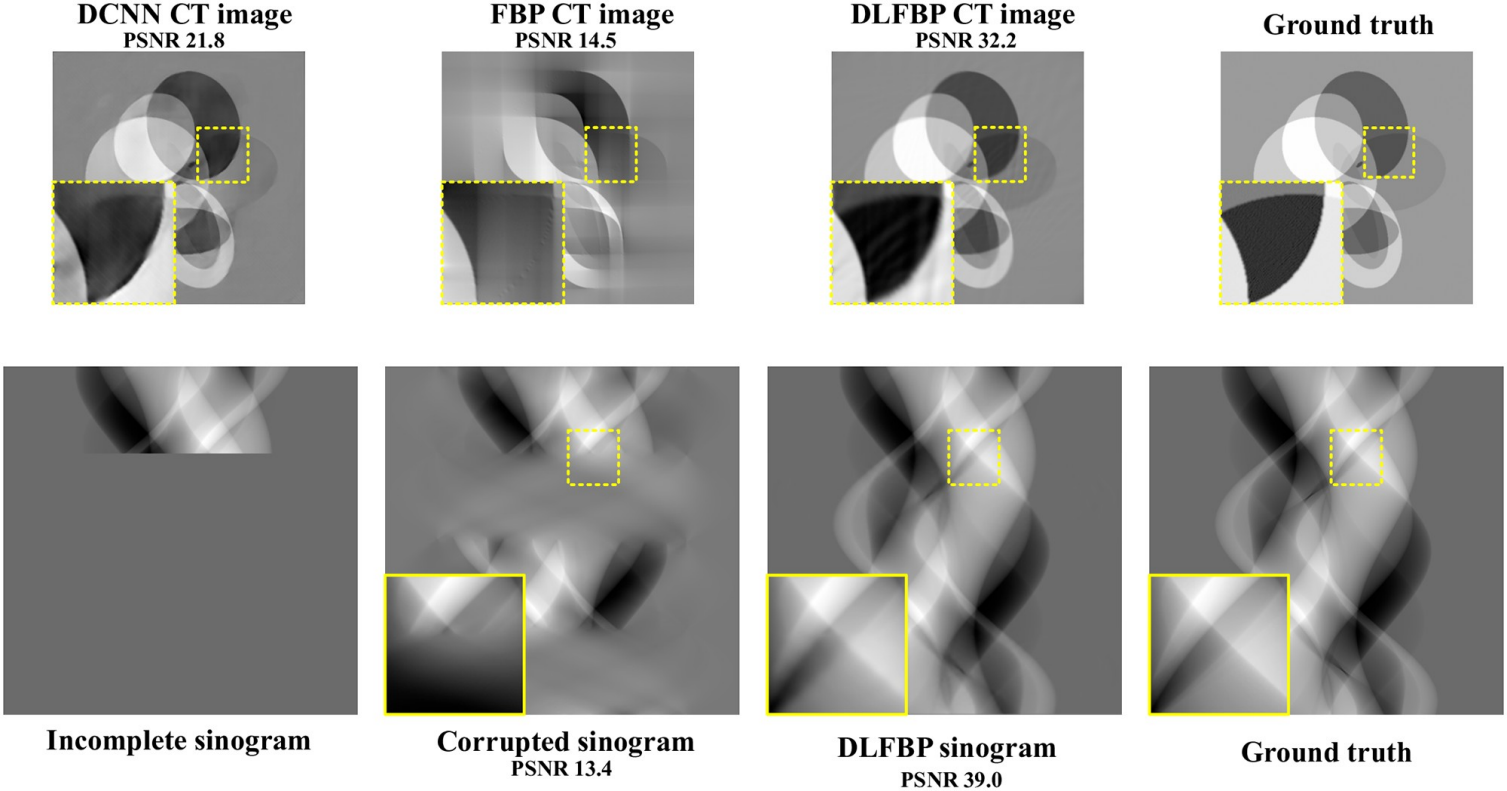
The limited-angle results of one synthetic phantom with the projections within the angular range [0 90°] by using DCNN [32], FBP and DLFBP. The incomplete singoram has a size of 180 × 731 pixels. The corrupted sinograms generated by the forward projection have a size of 720 × 731 pixels. All CT images have a size of 512 × 512 pixels after cutting off the surrounding blank region. Some regions of these images, indicated by the yellow box, are enlarged for better visualization.

**Fig 10:**
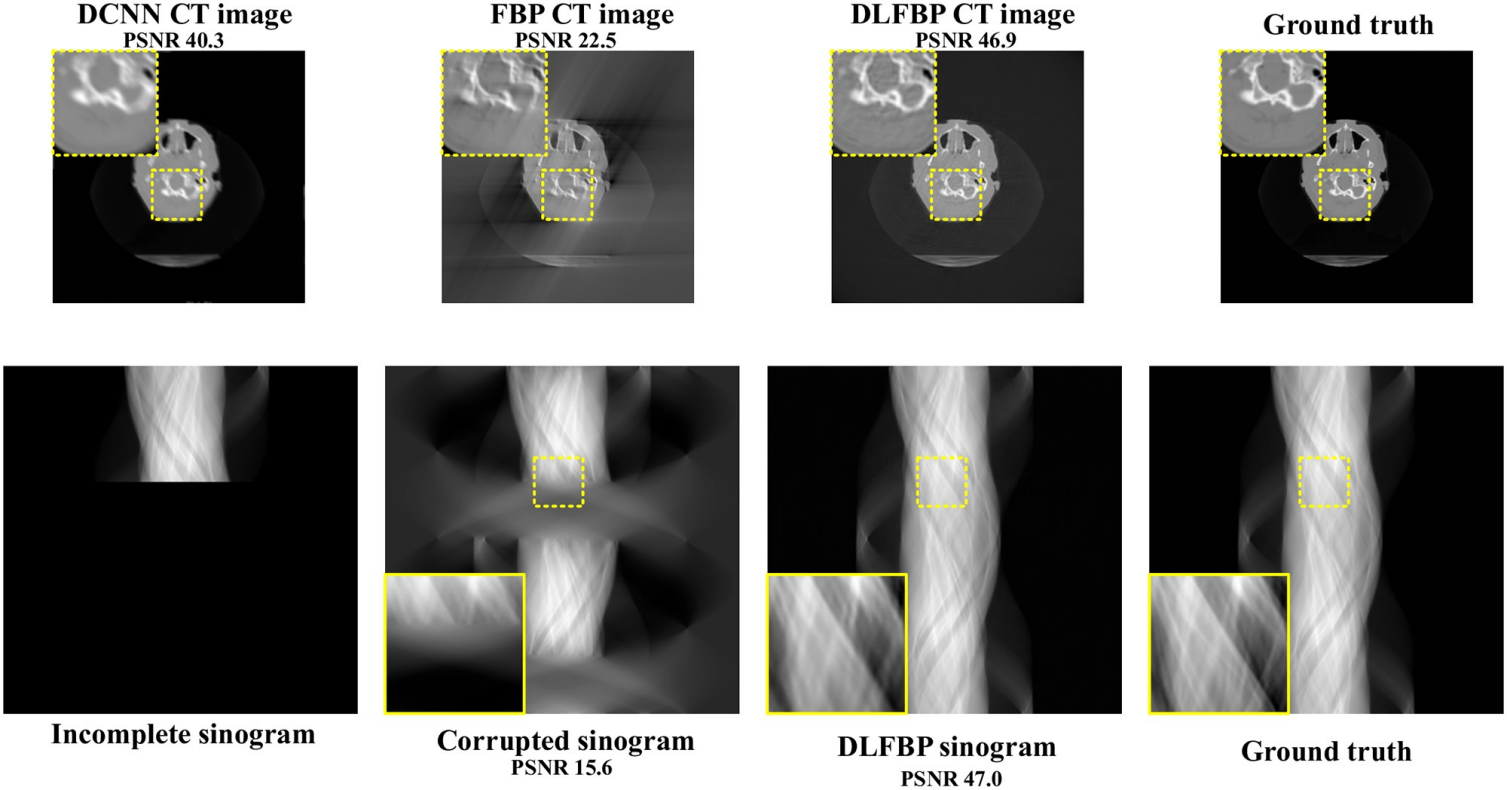
The limited-angle results of one head slice with the projections within the angular range [0 120°] by using DCNN [32], FBP and DLFBP. The incomplete singoram has a size of 240 × 731 pixels. The corrupted sinograms generated by the forward projection have a size of 720 × 731 pixels. All CT images have a size of 512 × 512 pixels after cutting off the surrounding blank region. Some regions of these images, indicated by the yellow box, are enlarged for better visualization.

**Fig 11:**
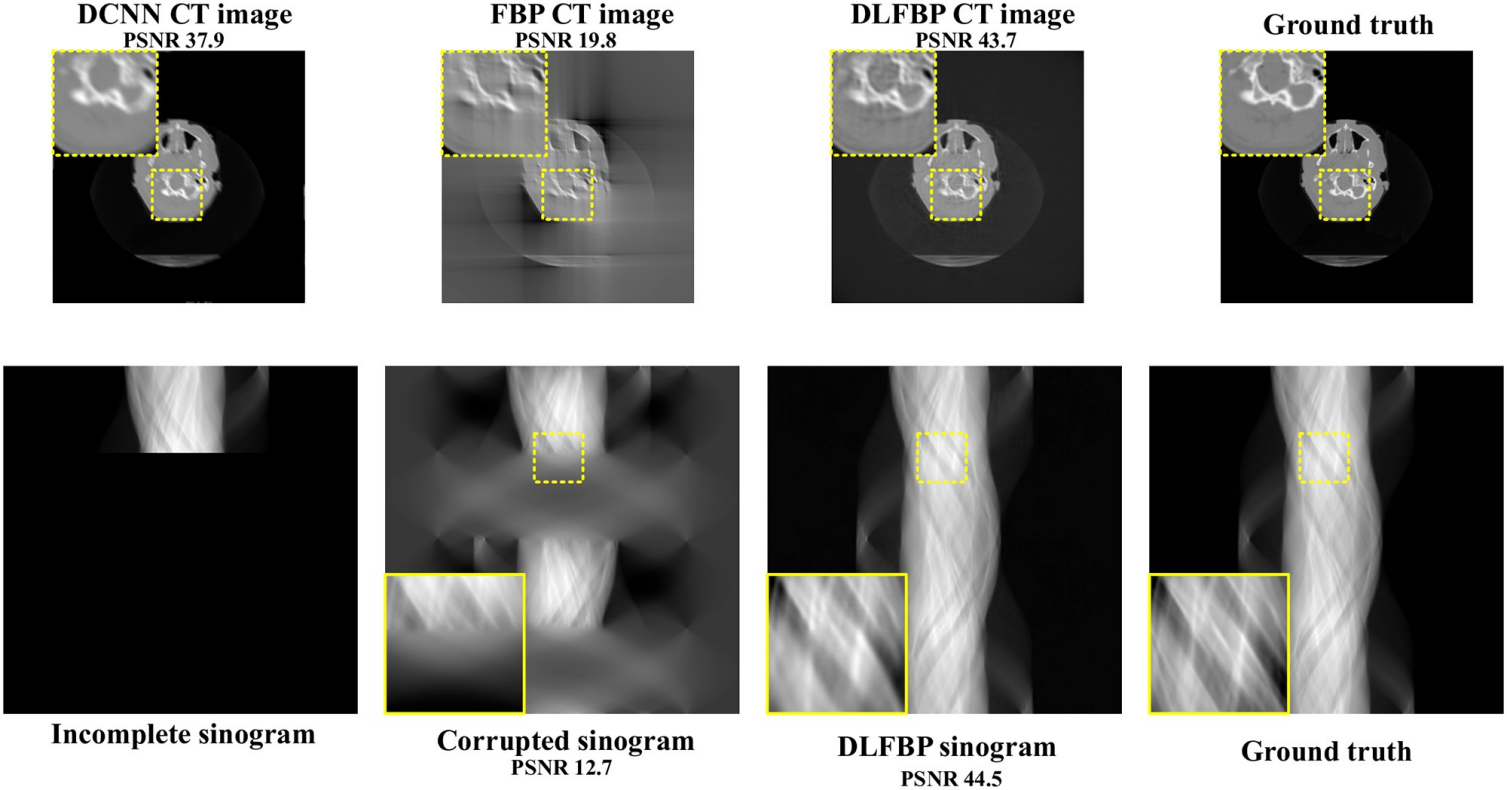
The limited-angle results of one head slice with the projections within the angular range [0 90°] by using DCNN [32], FBP and DLFBP. The incomplete singoram has a size of 180 × 731 pixels. The corrupted sinograms generated by the forward projection have a size of 720 × 731 pixels. All CT images have a size of 512 × 512 pixels after cutting off the surrounding blank region. Some regions of these images, indicated by the yellow box, are enlarged for better visualization.

**Fig 12:**
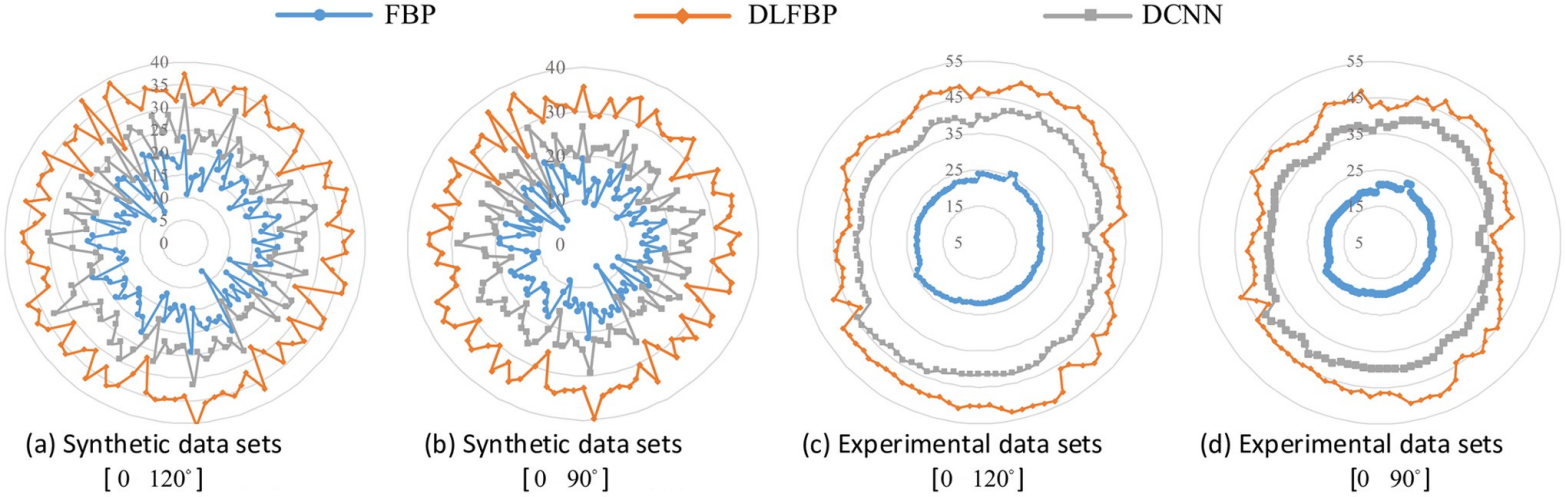
The PSNR values of the limited-angle CT images. The blue points with circle masks present the PSNR values of the CT images reconstructed by FBP. The orange points with rhombus masks present the PSNR values of the results by DLFBP. The gray points with square masks present the PSNR values of the results by DCNN [32].

Figs 8 and 9 present the limited-angle results of one of the 100 synthetic phantoms for testing. Figs 10 and 11 present the sparse-view results of one of the 100 head slices for testing. They correspond to the cases with the projections within the angular range [0 120°] and [0 90°], respectively. Their incomplete singorams have sizes of 240 × 731 pixels and 180 × 731 pixels, respectively. All the corrupted sinograms generated by the forward projection have a size of 720 × 731 pixels. All the reconstructed CT images have a size of 512 × 512 pixels after cutting off the surrounding blank region. In order to show the most obvious difference among each group of images, some regions of interest (ROIs) in these images, indicated by the yellow boxes, are enlarged for better visualization. Fig 12 shows the radar maps of the PSNR values of all the 100 synthetic and experimental images for limited-angle CT testing. Table 3 lists the corresponding aPSNR values.

**Table 3:** aPSNR values of limited-view results.

Cases		Corrupted Sinogram	DLFBP Sinogram	FBP CT Image	DCNN CT Image	DLFBP CT Image
Synthetic aPSNR(dB)	[0 120°]	16.27	39.74	16.99	24.12	33.78
	[0 90°]	13.52	38.99	14.60	21.97	32.38
Experimental aPSNR(dB)	[0 120°]	15.56	47.03	22.55	40.25	46.93
	[0 90°]	12.68	44.50	19.83	37.88	43.69

The images in Figs 8–11 and the values in Fig 12 and Table 3 show that DLFBP is effective to suppress the artifacts in limited-angle CT. Moreover, DLFBP recovers more inner structure details and the edge is much more sharper than DCNN.

## Discussion

Down-sampling with convolution filters plays an important role for the improvement of the image quality in the proposed framework. In conventional neural networks, max-pooling are usually adopted to implement down-sampling. They run fast, but the generated maximum value may not match the true one. In contrary, the coefficients in the convolution filters are determined by training the network with the training data set and better results can be provided by this operation.

In order to investigate the effect on learning accuracy from the down-sampling methods, the above experiments are repeated with max-pooling. Table 4 lists the corresponding aPSNR values. In any case, the aPSNR value of the convolution filter down-sampling is always larger than that of max-pooling. Obviously, the former performs better.

**Table 4:** aPSNR values of different down-sampling methods.

Cases			DLFBP Sinogram			DLFBP CT Image		
			Max-pooling	Mean-pooling	Strided-Conv.	Max-pooling	Mean-pooling	Strided-Conv.
Sparse-view	Synthetic aPSNR(dB)	90 views	52.57	52.86	53.29	38.50	37.42	39.01
		60 views	46.40	45.87	46.47	34.97	35.21	35.52
	Experimental aPSNR(dB)	90 views	52.54	51.21	53.15	39.67	39.10	40.22
		60 views	48.33	48.62	49.30	36.54	35.44	37.21
Limited-view	Synthetic aPSNR(dB)	[0 − 120°]	38.49	38.35	39.74	33.01	33.65	33.78
		[0 − 90°]	38.23	38.54	38.99	31.98	32.14	32.38
	Experimental aPSNR(dB)	[0 − 120°]	46.54	46.87	47.03	45.32	45.11	46.93
		[0 − 90°]	43.21	44.43	44.50	42.88	43.20	43.69

## Conclusion

In this paper, we reported a deep learning reconstruction framework for incomplete data CT. It is based on the deep learning technique and classical FBP reconstruction algorithms. All network parameters are not determined manually but by training in the domain of sinograms. It has been validated by the sparse-view and limited-view CT reconstruction with synthetic and experimental data sets. It provides a possible image reconstruction resolution for X-CT with incomplete data.

In the proposed framework, U-net neural network is adopted to train the incomplete sinograms. The learning accuracy and efficiency is limited by the characteristics of U-net. In the future, this framework could be improved further by replacing U-net with more advanced learning models.
